# Pesticides and environmental injustice in the USA: root causes, current regulatory reinforcement and a path forward

**DOI:** 10.1186/s12889-022-13057-4

**Published:** 2022-04-19

**Authors:** Nathan Donley, Robert D. Bullard, Jeannie Economos, Iris Figueroa, Jovita Lee, Amy K. Liebman, Dominica Navarro Martinez, Fatemeh Shafiei

**Affiliations:** 1 Center for Biological Diversity, Portland, OR USA; 2 Texas Southern University, Houston, TX USA; 3 Farmworker Association of Florida, Apopka, FL USA; 4 Farmworker Justice, Washington, DC USA; 5 Advance Carolina, Raleigh, NC USA; 6 Migrant Clinicians Network, Salisbury, MD USA; 7 Northwest Center for Alternatives to Pesticides, Eugene, OR USA; 8 Spelman College, Atlanta, GA USA

**Keywords:** Pesticides, Agrochemicals, Racism, Classism, Environmental justice, Regulation, Farmworkers, Worker safety, Children’s health

## Abstract

Many environmental pollutants are known to have disproportionate effects on Black, Indigenous and People of Color (BIPOC) as well as communities of low-income and wealth. The reasons for these disproportionate effects are complex and involve hundreds of years of systematic oppression kept in place through structural racism and classism in the USA. Here we analyze the available literature and existing datasets to determine the extent to which disparities in exposure and harm exist for one of the most widespread pollutants in the world – pesticides. Our objective was to identify and discuss not only the historical injustices that have led to these disparities, but also the current laws, policies and regulatory practices that perpetuate them to this day with the ultimate goal of proposing achievable solutions. Disparities in exposures and harms from pesticides are widespread, impacting BIPOC and low-income communities in both rural and urban settings and occurring throughout the entire lifecycle of the pesticide from production to end-use. These disparities are being perpetuated by current laws and regulations through 1) a pesticide safety double standard, 2) inadequate worker protections, and 3) export of dangerous pesticides to developing countries. Racial, ethnic and income disparities are also maintained through policies and regulatory practices that 4) fail to implement environmental justice Executive Orders, 5) fail to account for unintended pesticide use or provide adequate training and support, 6) fail to effectively monitor and follow-up with vulnerable communities post-approval, and 7) fail to implement essential protections for children. Here we’ve identified federal laws, regulations, policies, and practices that allow for disparities in pesticide exposure and harm to remain entrenched in everyday life for environmental justice communities. This is not simply a pesticides issue, but a broader public health and civil rights issue. The true fix is to shift the USA to a more just system based on the Precautionary Principle to prevent harmful pollution exposure to everyone, regardless of skin tone or income. However, there are actions that can be taken within our existing framework in the short term to make our unjust regulatory system work better for everyone.

**Supplementary Information:**

The online version contains supplementary material available at 10.1186/s12889-022-13057-4.

## Introduction

Pesticides have been used for thousands of years – with the first recorded pesticide ingredient, elemental sulfur, used over 4000 years ago in Mesopotamia [1]. As civilizations grew, so did the desire for easy ways to facilitate food production, prevent disease and manage nuisances. From ancient Egypt’s divination of cats as representations of gods and protectors of the home (quite adept at rodent control), to the “aim and spray” bottles that are found on store shelves today, modern society’s comfort with, and use of pesticides, has rapidly evolved.

A pesticide is anything that is intended to prevent, destroy, repel, or mitigate any pest [2]. This catchall term includes insecticides, herbicides, fungicides, bactericides, and rodenticides, among others. While typically thought of as a chemical component that is manufactured in a facility, the term “pesticide” can also encompass living organisms and management practices that seek to restore balance to an unhealthy system.

Commonly overlooked, the largest and most effective pest controller is nature itself. Traditional Ecological Knowledge (TEK) is an ever-evolving knowledge acquired by Indigenous and local peoples over thousands of years through direct relationship and connection with the land and surrounding environments [3]. The colonization of North America, known by some Indigenous people as Turtle Island, saw the brutal extermination of Native Americans and violently stolen land. Along with the loss of life, culture and TEK came a shift in ideologies that valued capital wealth, control, and expansion over balance and co-existence with land and people.

Hundreds of years would pass before the infamous pesticide, DDT, became a household name and ushered in an era of massive use of chemical pesticides in our daily lives [4]. During this ensuing time-period, structures of racism and class discrimination were erected in the USA through the systematic oppression and exclusion of BIPOC communities and people with lower socioeconomic status. This structural racism and classism, defined here as a system brought about by historical, institutional, cultural, or behavioral societal actions that routinely disadvantage, harm and cumulatively oppress BIPOC and/or people of low-income or wealth, has led to significant disparities in exposure to many pollutants that can lead to premature death or chronic disease [5–7].

Nearly 90% of pesticide use in the USA is in the agricultural sector, making agricultural laborers or farmworkers and their families particularly vulnerable to the effects of these dangerous chemicals [8]. Agricultural work in the USA was founded upon exploitative, dehumanizing mechanisms meant to reinforce white supremacy and prevent upward mobility of people of color. From the abhorrent use of African slave labor on Southern plantations and the subsequent practice of sharecropping and indentured servitude to the exploitation of Asian immigrants to do low-wage farm work along the West coast, racist agrarian structures are as old as modern agriculture itself [9, 10].

Just as chemical-intensive agriculture was becoming commonplace in the mid-twentieth century, the Bracero Program was implemented in the USA to facilitate the use of low-paying Mexican immigrant labor to fill agricultural positions left vacant during World War II [9, 11]. This further perpetuated a racial caste system in which wealthy, mostly white landowners profited from physically demanding, dangerous work done by people of color. Since the end of the Bracero Program, most labor and occupational safety laws have specifically excluded agricultural workers and, to this day, agricultural workers still have fewer protections than most other occupations in the USA [12].

Today 83% of farmworkers identify as Hispanic or Latinx [13]. The average annual income for a farmworker is less than $20,000 a year and one third of farmworkers had family incomes below the federal poverty line [13]. Upward mobility in agriculture is essentially nonexistent, as federal policies and racist lending practices have largely been responsible for 98 and 94% of all U.S. farmland being owned or operated by whites, respectively [9]. All these policies combined have all but ensured that BIPOC and people of low-income or wealth working in agriculture will consistently be the ones that bear the brunt of pesticide exposure in the fields.

In addition to disparate exposures at the workplace, racist and classist structures have led to disparate potential for exposure to harmful pesticides in or near people’s homes as well. This has ultimately led to many BIPOC and people of lower socioeconomic status being cordoned off into undesirable places within cities or rural areas that have poorer living conditions and very little political clout.

The 1800s saw overtly racist laws like the Indian Removal Act and Dawes Act that sought to erase Indigenous sovereignty and partition Indigenous people into small tracts of undesirable land. This was followed by the widespread use of eminent domain for “economic development,” racially-motivated zoning ordinances, and the practice of “redlining” in the early 1900’s that further partitioned and isolated BIPOC and communities of low income and wealth to areas that would receive less economic and social investment and ultimately deteriorate while other areas thrived [14]. More recent calls for “urban renewal” and subsequent gentrification has often further solidified these trends [15].

With regards to pesticide exposure, the consequences are two-fold. One is that these communities can often end up being located near toxic waste sites, including Superfund sites that contain legacy pesticide contamination, and are also directly targeted for new large-scale industrial chemical manufacturing and waste sites [16]. Most pesticides are synthetic chemicals that must be manufactured or synthesized in a facility. Polluting manufacturing facilities tend to be built in lower income communities with a higher proportion of people of color or in neighborhoods that were already in the process of transitioning to that end [17, 18]. Furthermore, hazardous facilities in lower income areas tend to invest less in pollution reduction than those in higher income areas [19].

Another consequence is that as housing structures in these communities deteriorate due to lack of resources and investment – coupled with often crowded living conditions in public or low-income housing – the heavy use of pesticides is often employed as a short-term fix for chronic pest problems. For example, in subsidized, public housing developments in New York state, 33% of residents reported applying pesticides indoors at least once per week [20]. This varied dramatically by housing density, with nearly *half* of residents in higher density public housing applying pesticides indoors at least *once per week* [20].

In this paper we review the scientific literature and publicly available datasets to determine the extent to which these historical injustices have led to disproportionate exposures and harms to low-income communities and people of color from pesticides. This analysis demonstrates that pesticide exposure and harm often fall upon racial, ethnic, and socioeconomic lines in the USA. While structural racism and classism have likely played an enormous role in shaping this trend, the objective of our study was to explore the current laws, policies and practices in the US government that are facilitating this disturbing trend and propose ways in which these institutional failings can begin to be rectified.

## How disproportionate pesticide impacts are realized

### Pesticide production

It is well-established that chemical manufacturing, storage, and waste affect BIPOC and impoverished communities more than the general population [21, 22].

An analysis of nine U.S. cities and counties that had high numbers of hazardous chemical facilities found that people who lived within three miles of those facilities were disproportionately African American or Latinx and living in poverty compared to the city or county as a whole [23]. Similar findings have been found on a national level, where African Americans and Latinxs living below the poverty line were more than twice as likely to live within a mile of a hazardous chemical facility [24]. The disproportionate exposure of low income and BIPOC communities to polluting industrial facilities is even more pronounced when analyzing those facilities that release the most harmful pollutants [25]. These polluting facilities also fail to create meaningful job opportunities for the members of the community that they are harming, further perpetuating the detriment to those who live nearby [26].

One of the most infamous industrial facility disasters in the world happened in 1984 in Bhopal, India, where a pesticide manufacturing facility exploded and covered the nearby poverty-stricken community in a toxic gas that ultimately killed thousands of people and injured over half a million [27]. In 2008, Bhopal’s sister facility in Institute, West Virginia – which used many of the same dangerous ingredients to manufacture pesticides – exploded, killing two people and blanketing the nearby community in dense smoke [28]. This was in an area of the state that had a 54% Black population compared to the state average of 3.6% and an average per-capita income that was two-thirds of the average in the surrounding counties [29, 30]. A Superfund site in Louisville KY, formally the Black Leaf chemical facility, which manufactured DDT and other pesticides, left widespread contamination in the surrounding area where 44% of people live below the poverty line and 84% of residents identify as Black compared with 16 and 8% in the state as a whole, respectively [31–34]. Another Superfund site, the former pesticide manufacturing facility United Heckathorn, heavily contaminated the harbor in the nearby city of Richmond, CA – where 84% of residents are people of color [35, 36].

**Fig. 1:**
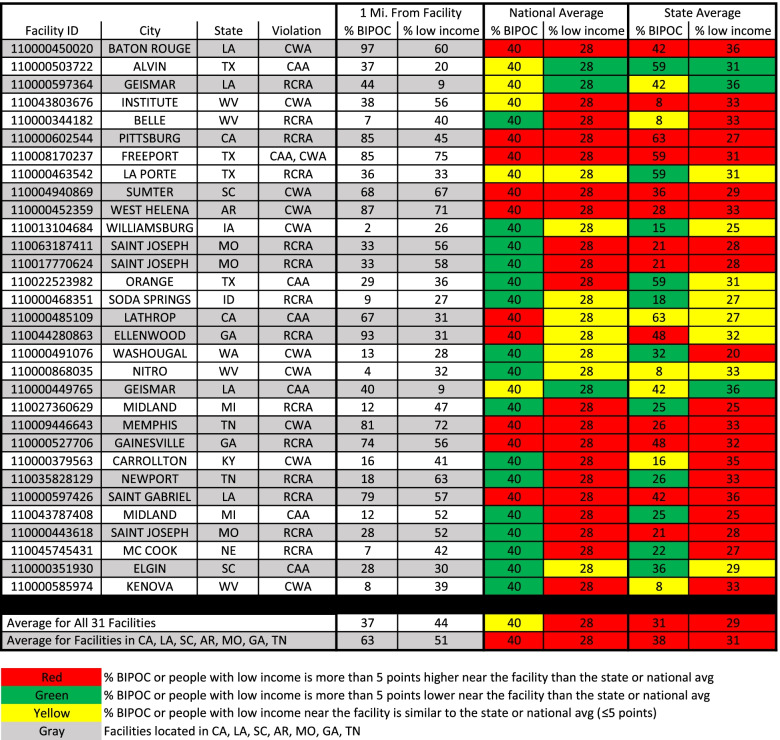
The % BIPOC and % Low-income Population that Reside Near Pesticide Manufacturing Facilities that Have Violated Environmental Laws Compared to National and State Averages. The first column gives the Facility ID as found in EPA’s Enforcement and Compliance History Online (ECHO) database. The second and third columns provide the city and state the facility is located in. The fourth column indicates the environmental law(s) that the facility has violated: Clean Water Act (CWA), Clean Air Act (CAA), or Resource Conservation and Recovery Act (RCRA). The fifth column provides the percent of people within one mile of the facility who do not identify as non-Hispanic, white (for the purposes of this Figure we have designated this population as Black, Indigenous and People of Color (BIPOC)). The sixth column provides the percent of people within one mile of the facility that have incomes below 200% of the federal poverty level. Columns 7–8 and columns 9–10 provide the national and relevant state averages of the percent of people who do not identify as non-Hispanic, white or have incomes below 200% of the federal poverty level. The bottom two rows compile the averages for each column for all facilities and facilities in California, Louisiana, South Carolina, Arkansas, Missouri, Georgia, and Tennessee

As of November 2021, there were 31 pesticide manufacturing facilities in the USA that the United States Environmental Protection Agency (EPA) had deemed in “Significant Violation” of bedrock environmental laws, including the Clean Air Act (CAA), Clean Water Act (CWA), and the Resource Conservation and Recovery Act (RCRA) (Additional file 1). An analysis of the demographics around these polluting facilities identified stark differences with state and national averages. An average of 44% of residents within one mile of these 31 pesticide manufacturing facilities had incomes less than two times the federal poverty level, compared to the national average of 28% and the relevant state average of 29% (Fig. 1).

The racial and ethnic demographics around these manufacturing facilities are more variable. Overall, there is little difference between the average percent BIPOC population within one mile of these facilities and the national average (Fig. 1). However, this gap widens when comparing to the relevant state average (37% BIPOC near facility compared to a 31% state average). Further examination of the data revealed significant variability from site to site, with about half of facilities having a higher BIPOC population within one mile of the facility and the other half having a lower BIPOC population nearby. The racial and ethnic variation appears to be largely regional, as California and many Southern states harbor the highest number of facilities in predominantly BIPOC neighborhoods, averaging a 63% BIPOC population within one mile of a facility compared to a 40 and 38% national and relevant state average, respectively (Fig. 1).

Three of the 31 pesticide manufacturing facilities are located in St. Joseph, MO and were recently ordered by a federal judge to be transferred to a third party to oversee their operations after thousands of containers of hazardous waste, stored in rusted or leaking containers, were found in dilapidated buildings that were in danger of collapse [37]. Lawsuits from federal and state governments allege that rainwater had been mixing with pesticide waste in the containers – ultimately leaking into the sewer system and nearby Missouri river [38]. The average “% BIPOC” and “% low-income” populations within one mile of these three facilities is 31 and 55% compared to the state average of 21 and 28%, respectively.

This indicates that pesticide manufacturing facilities in the USA that are in significant violation of bedrock environmental laws are disproportionately located in areas where a higher proportion of residents have low incomes. There is regional variation in whether these facilities are in areas with a higher BIPOC population – with this overwhelmingly being the case in California and many Southern states, but not elsewhere in the country.

### Pesticide use

#### Exposure

Worldwide, the burden of higher pesticide exposure is typically carried by the poorest and most vulnerable to exploitation [39, 40]. This is also the case in the USA, where exposure to pesticides correlates strongly with race, ethnicity, and socioeconomic status. Here we focus on the general trends in exposure to different subpopulations in the USA and further discuss specific demographic groups that are disproportionately bearing the societal burdens of pesticide use.

##### General trends

Researchers at the California EPA found that pesticide use was the pollution burden that showed the greatest racial, ethnic and income disparities in the state – disproportionately imposing more of a hazard than multiple air pollutants and other toxic releases [41]. The authors found that almost all pesticide use in the state occurs in the 60% of California zip codes that have the highest percentage of people of color. Others have found that over half of the glyphosate used in California was applied in the state’s eight most impoverished counties, where 53% of residents identified as Hispanic or Latinx compared to the state average of 38% [42]. In 2019, more than eight million pounds of pesticides linked to childhood cancers were used in the 11 California counties that had a majority Latinx population (>50%), resulting in 4.2 pounds of these pesticides used per person [43]. This contrasts sharply with the 770,000 pounds of these same pesticides used in the 25 California counties with the fewest Latinx residents (<24%), resulting in 0.35 pounds of these pesticides used per person [43]. Both groups of counties have comparable land area and population.

This is the case nationally as well, as African Americans and Mexican Americans had higher concentrations of pesticide biomarkers in their blood or urine than non-Hispanic whites who don’t live in poverty [44]. Similarly, biomarkers of pesticide exposure showed the greatest disparity between white women and women of color than 16 other chemical groups tested [45]. A U.S. Centers for Disease Control and Prevention (CDC) study found that metabolites of certain legacy pesticides were higher in Mexican Americans and African American women above the age of 40 than in whites [46]. The costs and disease burden associated with exposure to organophosphate pesticides were shown to be disproportionately borne by those who identify as non-Hispanic Black or Mexican American than non-Hispanic white [47].

**Fig. 2:**
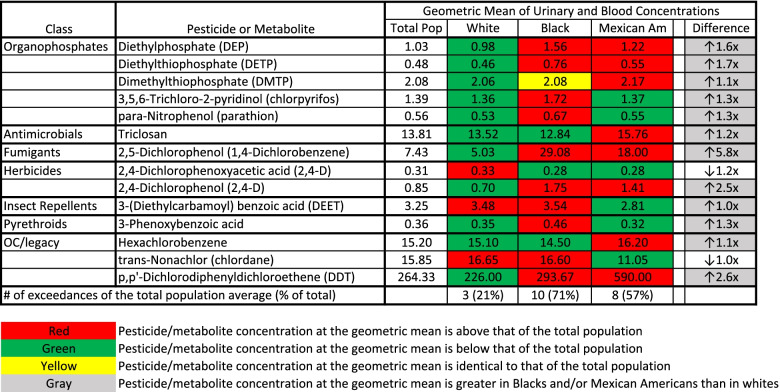
Average Urinary or Blood Pesticide/metabolite Concentrations in People of Various Demographic Groups in the USA. The first column identifies the class of the pesticide/metabolite. The second column identifies the specific pesticide/metabolite that was analyzed. The third, fourth, fifth, and sixth columns contain the geometric mean of the urinary or serum concentrations of each pesticide/metabolite for the total population (Total Pop), whites, Blacks and Mexican Americans (Mexican Am), respectively. All values are urinary concentrations (non-creatinine adjusted) in μg/L for all pesticide/metabolite classes except “OC/legacy.” For the “OC/legacy” pesticide/metabolite class, values are serum concentrations in ng/g of lipid. The last column is the fold change between the pesticide/metabolite concentration in whites and the demographic group with the highest pesticide/metabolite concentration. The last row indicates the total number (and % of total) of pesticides/metabolites for which the concentration in the demographic group exceeded that of the total population

To analyze a wider variety of pesticides across a national scale, we reviewed data collected by the CDC for the Fourth National Report on Human Exposure to Environmental Chemicals (Additional file 1). This report has information on a wide range of pesticides and pesticide metabolites that have been monitored in the blood and urine of a nationally representative sample of the U.S. population between the years of 1999–2016. Of 14 pesticides/metabolites that were found in high enough concentrations to identify a geometric mean for the three analyzed demographic subgroups (non-Hispanic white, non-Hispanic Black and Mexican American), only 3 (21%) were found in non-Hispanic whites at levels higher than the average for the total population (Fig. 2). In contrast, mean urinary and serum concentrations were higher for 8 of 14 (57%) and 10 of 14 (71%) pesticides/metabolites in Mexican Americans and non-Hispanic Blacks compared to the national average, respectively (Fig. 2). Non-Hispanic Blacks or Mexican Americans had higher average concentrations than non-Hispanic whites for 12 of the 14 pesticides/metabolites analyzed.

**Fig. 3:**
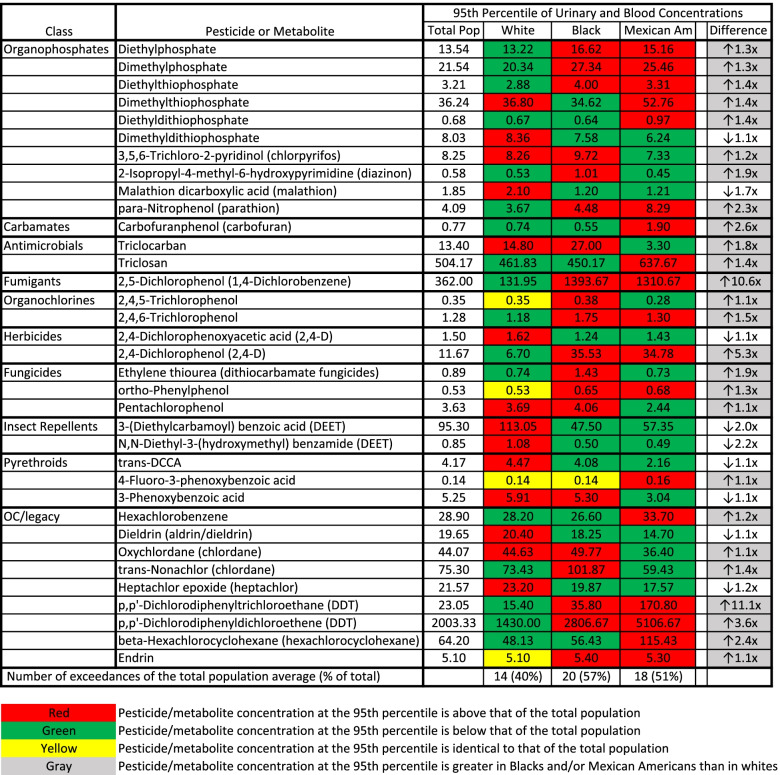
High-end Urinary or Blood Pesticide/metabolite Concentrations in People of Various Demographic Groups in the USA. The first column identifies the class of the pesticide/metabolite. The second column identifies the specific pesticide/metabolite that was analyzed. The third, fourth, fifth, and sixth columns contain the 95th percentile of the urinary or serum concentrations of each pesticide/metabolite for the total population (Total Pop), whites, Blacks and Mexican Americans (Mexican Am), respectively. All values are urinary concentrations (non-creatinine adjusted) in μg/L for all pesticide/metabolite classes except “OC/legacy.” For the “OC/legacy” pesticide/metabolite class, values are serum concentrations in ng/g of lipid. The last column is the fold change between the pesticide/metabolite concentration in whites and the demographic group with the highest pesticide/metabolite concentration. The last row indicates the total number (and % of total) of pesticides/metabolites for which the concentration in the demographic group exceeded that of the total population

A similar trend was apparent with the highest exposed individuals from each demographic subgroup. Of 35 pesticides/metabolites where concentrations at the 95th percentile were reliably identified, the highest exposed non-Hispanic whites, Mexican Americans, and non-Hispanic Blacks exceeded the 95th percentile for the total population 40, 51 and 57% of the time, respectively (Fig. 3). Non-Hispanic Blacks or Mexican Americans had higher concentrations at the 95th percentile than non-Hispanic whites for 26 of the 35 pesticides/metabolites studied.

This indicates that not only do non-Hispanic Blacks and Mexican Americans tend to have higher average urinary and blood levels of many pesticides, but that the highest exposed individuals within these demographic groups are more likely to be exposed to higher quantities than the highest exposed non-Hispanic whites.

Altogether, the available literature and data suggest that BIPOC and people living in poverty are generally exposed to higher levels of pesticides than the total population at large. This presents a serious environmental justice issue that must be addressed.

##### Children

In California, almost three out of every four children with the highest potential for exposure to pesticides at school were non-Anglo [48]. An analysis of 15 agricultural counties in California found that children identifying as Hispanic were 46% more likely than white children to go to school within a quarter mile of where pesticides of human health concern were used [49]. Hispanic children were also 91% more likely than white children to attend school where the highest amount of pesticides of human health concern were used nearby [49]. In Washington state, more than half of students who attended school in counties with the most agriculture did not identify as white compared to a 31% student average in the state [50]. Eight-year-old Latinx children in low-income households in North Carolina were exposed to an average of 5.7 different pesticides in a three-month timeframe, with the specific pesticide exposures differing whether they lived in a rural or urban area [51].

Children are more susceptible to the effects of environmental toxins like pesticides because they are still in a developmental stage of life. With children of color more likely to be exposed to pesticides, they are not only more susceptible, but more vulnerable to pesticidal harm. Children of color are therefore the most vulnerable of any vulnerable population subgroup and will often be the most at-risk population.

##### Urban and low-income housing

Pesticide use is often heavy in inner-city housing due to the age of the structures, inadequate maintenance and often crowded living conditions [52]. Residential pesticide use tends to increase with higher housing density and pesticides were found to be widely used in low-income public housing in New York state – where 80% of facilities applied pesticides inside apartments and in common areas on a regular basis [20]. A study of public housing facilities in Boston, MA, where 98% of residents identified as Hispanic or Black, detected at least two pesticides in all 42 units analyzed and at least six in the majority of units [53]. Eighty five percent of pregnant African American and Dominican women in New York City reported using pesticides in their residence and 83% had at least one pesticide in umbilical cord samples at birth [54]. Thirty percent of African American and Dominican mothers had at least eight pesticides detected in a home air monitoring study [55]. An analysis of seven pesticide biomarkers in women from Long Island, New York found that the average total pesticide concentration in breast adipose tissue was about 10% higher in Black women than white women [56].

The majority of a person’s life is often spent inside their home. Housing, therefore, represents a serious potential exposure pathway to many environmental justice communities in the USA. While some people are able to control environmental contaminants that enter their home to a certain degree, many do not have that luxury and are subject to the whims of what a landlord or management company decides to do (often without prior consent).

##### Farmworkers

Due to the nature of their work and where they live, farmworkers – and by extension their families – are thought to be the group of people most highly exposed to agricultural pesticides. Urinary analysis of nearly 200 farmworkers in North Carolina found that not only were they exposed to a wide array of chemical pesticides, but that re-exposure was constant throughout the year [57]. Similar findings in Idaho found insecticide and herbicide metabolites in the urine of Latinx farmworkers in every sample tested, even after pesticide spraying season was done [58]. Hispanic and Haitian female farmworkers in Florida were found to have much higher levels of urinary pesticide metabolites than a nationally-representative survey [59]. Farmworkers in Monterey County, CA had median urinary pesticide metabolite levels that were up to 395 times higher than a nationally-representative survey [60]. Pesticides and pesticide metabolites were found in dust samples in 85% of Washington farmworker homes and in the urine of 88% of young children with whom they lived, indicating that work exposure can often transfer to the home [61].

The vast majority of pesticide use in the USA is in agriculture and farmworkers have always been the most highly exposed group of people to agricultural pesticides. Many also reside in housing where residential pesticide use is high. While the nature of their work means that farmworkers will likely always have somewhat of a higher exposure to pesticides than the general population, the current disparities in the USA are far beyond what should ever be considered acceptable.

#### Effects

##### In the USA

Higher exposures of many pesticides at concentrations of human relevance are often associated with increased disease incidence [62, 63], and there is increasing evidence that the specific exposures that disproportionately burden BIPOC and communities of low income and wealth can lead to disproportionate levels of acute harm or disease.

There are major barriers in place that make it difficult to tie specific exposures of pesticides to specific harms, particularly to BIPOC communities and those living in poverty. Poison Control Center utilization is known to be much lower in BIPOC and low-income populations, making comparisons between different racial, ethnic and income demographics very difficult [64–66]. Correctly diagnosing illness from acute pesticide harm requires the harmed individual to have access to, and seek, medical treatment, which often doesn’t happen [67]. Furthermore, the physician (often un- or under-trained in this area) must also be able to correctly identify and diagnose the problem and report it [68]. Other significant barriers can lead to even greater underestimates of harm to seasonal and migrant laborers [69]. All these difficulties are compounded when it comes to chronic effects from long-term pesticide exposure that don’t have the same immediate temporal association with exposure that acute effects have.

Despite the enormous difficulties in tying pesticide exposure to harm in certain populations, pesticide exposure among low-income and BIPOC populations has routinely been associated with adverse health outcomes.

By extrapolating from hospital visits in California, the US EPA estimated that 10,000–20,000 agricultural workers (predominately Latinx) experience physician-diagnosed, acute illness each year in the USA due to pesticide exposure, and that number could be as high as 300,000 acute illnesses per year when accounting for workers who don’t seek care from a medical facility [70, 71]. Surveillance of occupational injuries in the state of Michigan found that people who identify as Hispanic are more likely to become ill due to pesticide exposure on the job than non-Hispanics [72]. Between 2007–2011, the rate of acute occupational pesticide-related illness and injury was 37 times higher for agricultural workers than for non-agricultural workers [73]. Occupational exposure to some agricultural pesticides is associated with an increased risk of breast cancer in California Latinx women [74]. Studies on Mexican American children in a farmworker community in California found that exposure to certain pesticides *in utero* or after birth was associated with negative effects on attention and neurological impacts that can affect cognitive and behavioral function [75, 76].

It’s not just on-the-job exposures that can result in harm. Multiple pesticides and pesticide metabolites were found at higher levels in non-Hispanic Black women than non-Hispanic white women, and those higher blood and urine concentrations in non-Hispanic Black women were found to have breast cancer associated biological activity [77]. Serum levels of two pesticide metabolites were associated with an increased risk of diabetes in an adult Native-American (Mohawk) population, while serum levels of another pesticide were associated with a decreased risk [78]. The association between serum levels of certain chlorinated pesticides and type 2 diabetes was stronger in people who do not identify as white than those that do [79]. A study on pregnant African American and Dominican women in New York City found that pesticide levels in cord plasma were negatively associated with fetal growth [80, 81]. A study on mothers and newborns from Cincinnati found that urinary maternal levels of organophosphate metabolites were more strongly associated with decreased birth weight among Black newborns than white newborns [82]. This same study also found that those urinary metabolites were associated with shorter gestation time only in white mothers and not Black mothers. Non-Hispanic and Hispanic whites were grouped together for this study and it’s been previously shown that similar metabolites in Latina women were associated with decreased gestational duration [83].

Attempts to pool cohorts from multiple epidemiological studies also identified some racial and ethnic heterogeneity among associations with pesticide exposure and various neurological and reproductive outcomes; with those who identify as Black or Hispanic showing stronger negative associations between pesticide exposure and certain negative effects compared to those who identify as white [84, 85].

Disproportionate pesticide exposures are often associated with human health harms in low-income and BIPOC communities in the USA, however the true scope of harm is often unknowable due to the inherent difficulties in documenting these harms in underserved and overburdened communities.

##### Internationally

While the focus of this study is the disproportionate pesticide impacts in the USA, it is important to understand that these issues exist across political boundaries. In fact, by being a major manufacturer and exporter of pesticides, the USA plays a role in how these impacts are realized abroad.

Surveys conducted across Africa, Asia and Latin America have found that people in farming communities often lack access to, or cannot afford, suitable Personal Protective Equipment (PPE) for pesticide application and subsequently suffer from headaches, nausea, dizziness, blurred vision and excessive sweating [86]. A recent study estimates that around 385 million cases of acute pesticide poisoning occur each year worldwide, with the majority of that harm occurring in developing countries [87]. A report for the World Health Organization and United Nations Environment Programme identified women and children as the most vulnerable to pesticide impacts worldwide [88].

If there is one constant we’ve identified with regards to pesticide exposure and harm, it is that the most vulnerable individuals and communities will routinely be the ones shouldering a disproportionate burden of the societal harm caused by pesticides.

## How disproportionate pesticide impacts are currently perpetuated

### Rooted in U.S. law, regulations, policies and regulatory practice

Below we discuss various aspects of the pesticide regulatory framework in the USA and how they function to maintain the status quo with regards to disproportionate pesticide impacts to environmental justice communities. This is not an exhaustive list, but areas where we believe have the most impact to on-the-ground communities. Each subsection identifies laws, regulations, policies and/or regulatory practices that are responsible for perpetuating disproportionate harm to people of color and low-income communities.

#### Double standard for pesticide safety

As the major pesticide law in the USA, the Federal Insecticide, Fungicide, and Rodenticide Act (FIFRA) controls the approval, sale, and distribution of pesticides. Together with the Federal Food, Drug and Cosmetic Act (FFDCA), which governs the allowable residues of pesticides on food, these two laws form the basis for pesticide regulation in the USA. Twenty-five years ago, Congress passed the Food Quality Protection Act of 1996 (FQPA), which amended FIFRA and the FFDCA [89]. Specifically, the FQPA put in place a new safety standard of a “…reasonable certainty that no harm will result…” to people exposed to pesticides through food and all other non-occupational exposure routes [89, 90]. However, all occupational pesticide exposures to people still default to the previous safety standard of no “…unreasonable risk to man or the environment, taking into account the economic, social, and environmental costs and benefits of the use of any pesticide…” [91].

In practice what this means is that for the general population, exposed mainly to pesticides through their diet, water and residential use, EPA takes a risk-only approach – approving a pesticide only if the agency determines that it will not result in significant harm. Yet for farmworkers and those exposed to pesticides mainly through their work, EPA takes a cost-benefit approach whereby harm to workers is allowed as long as the purported benefit of the pesticide, presumably to the grower, sufficiently offsets those harms.

Having two separate safety thresholds for different populations of people institutionalizes the practice of prioritizing some people’s lives over others and, by design, leads to enormous disparities in who is being harmed by pesticides. With the farmworker population overwhelmingly identifying as Hispanic or Latinx, this creates an enormous environmental justice issue.

The EPA seemingly recognizes this terrible double-standard, and in 2009 published a proposed policy document aimed at strengthening its occupational risk assessment entitled “Revised Risk Assessment Methods for Workers, Children of Workers in Agricultural Fields, and Pesticides with No Food Uses” [92]. This document identifies ways EPA can more closely align the occupational and non-occupational risk assessment, stating: “No scientific justification exists for distinguishing between otherwise identical exposures based on whether they occurred on-the-job or not” [92].

Following fierce opposition from the American Chemistry Council and the pesticide industry [93–95], this 12 year-old proposed policy still remains in draft form. While the EPA has implemented a few of the components of this draft policy already, the agency has made only minimal progress in implementing the more consequential proposed policy changes [96].

#### Inadequate worker protections from pesticides

In addition to a long and much broader history of farmworker “exceptionalism,” where farmworkers have consistently been excepted from basic labor rights, farmworkers also lack many basic occupational safety protections from pesticide exposure [12]. While most occupational sector safety standards are overseen by the U.S. Occupational Safety and Health Administration (OSHA), the agency has largely relegated the realm of agriculture to the EPA, which has since exerted its authority over pesticide worker safety with the Worker Protection Standard (WPS) regulation issued under FIFRA [12, 97]. The very fact that the agency in charge of approving pesticides is the same one that’s in charge of establishing and enforcing worker standards is troubling to say the least.

In 2015 the WPS was strengthened, providing further protections for farmworkers than what they had been afforded in the past [98]. Despite these improvements (some of which were targeted for removal in a subsequent rulemaking [99]), worker protections from pesticides remain grossly substandard.

Biological monitoring of workplace chemical exposures is common in many industries and OSHA has developed over 25 chemical standards that are to be used to screen workers that are exposed to hazardous substances as part of their work [100]. Yet despite farmworkers coming into constant, often daily, contact with chemical pesticides that are known to be harmful, there is no national requirement for employers to provide medical monitoring for farmworkers seeking to prevent chronic, harmful pesticide exposures. This is even more worrisome given that perceptions of pesticide exposure at the workplace don’t always correlate with actual exposure [59].

Some states, like California and Washington, have implemented biological monitoring programs for certain pesticide classes in an effort to protect farmworkers in those states [101, 102]. What these state programs have identified is cause for concern; in cases where pesticide exposure resulted in physiological effects to workers, many were not even the result of a violation of the WPS or the pesticide label, suggesting that following the directions on the label is not necessarily protective of pesticide harm [103]. Since some pesticide exposures can lead to adverse effects in the absence of readily noticeable symptoms [104], biological monitoring is absolutely necessary to prevent or reduce harm from chemical exposure.

While the WPS does provide some legal protections for farmworkers, lack of compliance monitoring and enforcement provides little incentive for employers to follow the rules. Nearly all workplace inspections are conducted by the states, leading to major inconsistencies from state to state. In 1998, five states conducted no workplace inspections for WPS compliance and 11 states conducted fewer than ten [105].

While these numbers have modestly improved since then, only a small minority of workplaces are inspected in any given year. Data from EPA’s ECHO database indicate that, for the most recent five years that data are available, just over 1% of pesticide-using agricultural operations were inspected for WPS violations (Table 1). This means that at the current rate of inspection it will take nearly 100 years to inspect all facilities that fall under the Standard. During this period the few inspections that were conducted found a considerable number of violations – there was an average violation rate of 49%, indicating that nearly one WPS violation was found for every two facilities that were inspected (Table 1). Despite the majority of violations being for highly consequential failures such as failure to provide pesticide safety training, failure to centrally post vital information about pesticide use on the premises, and failure to provide proper PPE, only about 19% of violations led to any action other than a warning (Table 1) [106].

**Table 1:** Worker protection standard compliance and violation enforcement from 2015–2019

Year	Total			No Action/	Enforcement			
	WPS	Facilities	Violations	Only	Action	Inspection	Violation	Enforcement
	Facilities	Inspected	Found	Warning	Taken	Rate	Rate	Rate
2019	304,106	3475	1903	1595	308	1.1%	54.8%	16.2%
2018	304,106	3774	2057	1676	381	1.2%	54.5%	18.5%
2017	304,106	3418	2296	1997	299	1.1%	67.2%	13.0%
2016	304,106	3320	1142	789	353	1.1%	34.4%	30.9%
2015	304,106	3557	1199	925	274	1.2%	33.7%	22.9%
5-yr Avg	304,106	3509	1719	1396	323	1.2%	49.0%	18.8%

WPS violations appear to be very common despite the low number of inspections that are conducted every year by the EPA, the states, and tribes. A near-50% violation rate is *very* high and indicates that a significant portion of the estimated 1.8 million workers and handlers who work in these facilities are not receiving legally-mandated protections from pesticides. Furthermore, 80% of violators don’t even receive a slap on the wrist after they are found to have violated the law. Without the prospect of facing any meaningful consequence, there is no deterrent for unscrupulous employers to follow the rules, which perpetuates exploitative working conditions.

#### Export of dangerous pesticides to developing countries

It’s been estimated that 385 million cases of unintentional, acute pesticide poisoning (UAPP) occur each year worldwide, with the greatest number of poisonings happening in developing regions of the world in southern and south-eastern Asia and east Africa [87]. FIFRA section 17 (a)(2) allows for the manufacture and export of pesticides to other countries that are not registered in the USA if certain labelling and notification requirements are met – this includes the export of pesticides that have never been approved in the USA or cancelled due to human health or environmental concerns [107]. The extent of the export of pesticides that are prohibited in the USA is substantial. An analysis of U.S. customs shipping records found that between 2001–2003 the USA exported nearly 28 million pounds of pesticides that were not allowed to be used in the country, averaging 13 tons/day [108]. This included many pesticides that the USA had banned due to human and environmental health concerns and others that were subject to regulation under international treaty, like dinoseb, mercury-based pesticides, endosulfan and pentachlorophenol [108].

In 2009 the EPA Office of the Inspector General (EPA-OIG) analyzed EPA’s compliance with FIFRA section 17(a). The EPA-OIG found that EPA does not ensure that an importing country is notified (as required by law) that a pesticide found to be harmful to human health – or a pesticide for which no EPA assessment had been conducted – is being exported to their country [109]. In fact, EPA notified the importing countries for only 3% of such pesticide exports in 2007, prompting the EPA-OIG to conclude that importing countries may not be aware of potential hazards associated with pesticides they import from the USA [109].

**Fig. 4:**
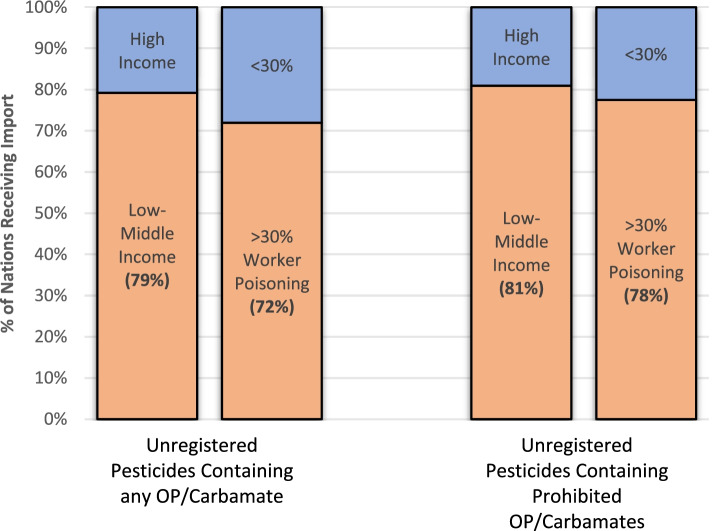
Export of Unregistered Pesticides to Different Nations Stratified by Wealth and % Workforce Poisoned. The first two bars represent the percentage of nations receiving import of unregistered pesticides containing any organophosphate (OP) or carbamate active ingredients from the USA. The first bar stratifies these nations by Gross National Income (GNI) – the two categories being high-income or low-to-middle income as defined by the World Bank. The second bar stratifies these nations by the percent of agricultural workers in each country that are estimated to experience an unintentional pesticide poisoning each year – the two categories being >30 and < 30%. The third and fourth bars represent the percentage of nations receiving import of unregistered pesticides containing prohibited organophosphate (OP) or carbamate active ingredients from the USA. The third and fourth bars are stratified identically to the first two bars. The only difference between “Unregistered Pesticides Containing any OP/Carbamate” and “Unregistered Pesticides Containing Prohibited OP/Carbamates” is that the former contain OP/Carbamates that are allowed for use in other, registered products in the USA while the latter contain OP/Carbamates that are completely banned for use in any product in the USA

Organophosphate (OP) and carbamate insecticides are known neurotoxins responsible for many pesticide poisonings around the world due to their high acute toxicity [110–112]. Between the years of 2015–2019, unregistered pesticide products containing 26 different OP or carbamate insecticides were manufactured or formulated in the USA for export (Additional file 1). These products were exported to 53 different nations, 79% of which are considered low-to-middle income countries (LMICs) by the Organisation for Economic Co-operation and Development (OECD), and eligible for financial development and welfare assistance (Fig. 4 and Additional file 1). Of the 42 nations that imported unregistered products containing OP/carbamate ingredients that are completely prohibited for use in the USA, LMICs made up 81% (Fig. 4).

Similar trends were identified when stratifying nations by how much of their agricultural workforce is estimated to be poisoned by pesticides each year. Seventy two percent of nations importing unregistered products that contain any OP/carbamate ingredients from the USA are estimated to have >30% of their agricultural workforce poisoned by pesticides each year (Fig. 4 and Additional file 1). That proportion increases to 78% of importing nations for unregistered products containing an OP/carbamate ingredient that is completely prohibited for use in the USA (Fig. 4).

Allowing the manufacture and export of pesticides that have been banned in the USA, or whose safety has not been properly vetted, not only puts at risk vulnerable people in other countries but also places a higher burden on fenceline communities in the USA that live near the polluting facilities that manufacture them.

#### Failure to implement executive order 12898

Executive Order 12898, “Federal Action to Address Environmental Justice in Minority Populations and Low-income Populations,” was signed by President Bill Clinton in 1994 to direct federal agencies to use existing laws to prevent BIPOC and low-income populations from being disproportionately burdened by the impacts of environmental pollutants [113]. Despite the clear intent of the order, and the clear potential for pesticides to disproportionately impact BIPOC and low-income communities, the EPA pesticide office has routinely failed to adequately implement this order more than 25 years after it was signed.

In multiple EPA-OIG investigations in 2004 and 2006, the watchdog agency found that EPA had not even implemented guidance for how the agency could begin to comply with the Order [114, 115]. Sixty percent of responding offices had not performed the necessary reviews required by the Order 12 years after it was signed and 87% said that management had not even requested such reviews be undertaken [115]. These general conclusions have been confirmed at EPA and other federal agencies by academic researchers [116, 117].

An analysis of final rule-making actions by the EPA between the years of 1994–2012 found that EPA overwhelmingly utilizes pro forma acknowledgement of EO 12898 that an agency action would have no impact on environmental justice communities [118]. This contrasts greatly with the miniscule number of “affirmative” uses of the Order in final rules where the action would have beneficial impacts on those same communities [118].

In our experience, pro forma acknowledgement of EO 12898 is standard practice at the EPA pesticide office, with all recent human health pesticide risk assessments containing the same boilerplate language that the EPA considered environmental justice concerns in its assessment by analyzing the dietary patterns of certain ethnic subgroups (some examples here [119–121]). Yet, other than analyzing some differing exposures via diet, there are no other analyses currently undertaken to quantify or mitigate higher exposures to BIPOC or low-income communities more than 25 years after such actions were required.^1^

#### Failure to account for unintended (off-label) pesticide use or provide adequate training and support

When faced with the decision of whether to approve a pesticide that can cause harm to people, the EPA will often impose use restrictions on the pesticide label, such as PPE requirements, meant to mitigate harm from the pesticide. These restrictions can range from relatively minor to excruciatingly complex, as evidenced by a recent 9th Circuit Court of Appeals ruling that a recent pesticide approval was unlawful, in part, because the label directions were impossible to follow [122].

Unintended or “off-label” pesticide use is common and can have tangible consequences [123]. For example, when three women who worked on the same farm during their pregnancies all gave birth to children with congenital anomalies, it was subsequently found that the farm they worked at failed to prevent entry into treated fields after pesticide spraying and that the pesticide label requirements were not followed [124].

EPA approves pesticides assuming that all pesticide label directions can and will be followed, yet that assumption is often at odds with reality. Five requirements must be met for a pesticide label to serve its intended function: 1) the user must have access to the label or the internet if the full label is too big to fit on the container, 2) the label must be in a language the user can understand, 3) the user must be literate, 4) the user must be able to understand the technical language in the label directions, and 5) the user must have the ability or support to implement the safety precautions (PPE, mixing instruments, etc.) [125].

These five requirements are often not met in the United States population, including in farmworkers. A study of binational farmworkers that mixed and loaded pesticides on US farms found that nearly a quarter used no protective equipment the last time they worked with chemicals [126]. A survey of Oregon farmworkers found that 61% had reported breathing in pesticides from the surrounding air, 39% had touched plants with pesticide residue, and over one third had been sprayed with pesticides directly from a plane or tractor – all scenarios the pesticide label is supposed to prevent [127]. A quarter of surveyed North Carolina farmworkers were asked by their employer to enter fields too soon after pesticides had been applied, in violation of the label [128].

Often, unintended pesticide use is due to a lack of training or support [68]. Anywhere from 14–65% of surveyed farmworkers across multiple states reported receiving no pesticide safety instruction by their employer [13, 128–131]. Of North Carolina farmworkers that did receive pesticide training, less than half fully understood it [129]. Few were provided PPE or safety equipment [128, 132, 133]. Despite only 28% of farmworkers reporting that they can read English “well,” it is still not required that pesticide companies provide pesticide labels in a language other than English [13, 134].

The EPA often recites the adage, “the label is the law.” Ignoring the reality on the ground that pesticides are widely used in a manner not in compliance with the label – regardless of what laws or regulations are in place to prevent it – ultimately disadvantages those who are suffering the burdens of those exposures the most.

#### Ineffective post-approval follow-up

New pesticides are often approved with just a handful of pesticide toxicity studies done by the pesticide companies seeking approval. While pesticide law requires the EPA to re-analyze the safety of pesticides every 15 years to incorporate new science and other information [135], in practice this effort is often marred by a lack of follow-up data on the most highly-exposed people and regressive practices that often prevent meaningful incorporation of high-quality epidemiological studies.

The U.S. government is estimated to undercount agricultural injuries by 70–95%, which is more than any other industry [136, 137]. The inherent difficulties in monitoring a workforce that is predominantly migrant and seasonal is exacerbated by an ineffective, underfunded system to monitor and compile incidents of harm. Pesticide incident reporting is overseen by states governed by a patchwork of laws and regulations that range from semi-robust to non-existent [138]. Most, if not all, are plagued by funding deficiencies and undercounting [138]. The federal government’s response to this was to develop a federal-state hybrid surveillance system called the Sentinel Event Notification System for Occupational Risk (SENSOR)-Pesticides program [139]. In the SENSOR-Pesticides program, 12 of the 50 states have historically agreed to submit information to the CDC in exchange for some federal funds [140]. Only seven of those states submit non-occupational pesticide related injuries [140]. In 2019–2020, only three states received federal support for participation in the program [141].

While the SENSOR-Pesticides program was an improvement upon the state-by-state approach and allowed the federal government to monitor trends and standardize incident collection protocols among participating states, it is not robust enough to adequately capture pesticide exposure incidents at the national level. In most states, occupational incident reporting is exclusively the responsibility of healthcare providers and those who have been poisoned [138]. Barriers, such as lack of health insurance, language access, transportation, availability during hours of facility operation, immigration status, and fear of retaliation or further oppression, prevent many farmworkers from seeking care at a medical facility or reporting poisonings even when their injuries are serious [67–69, 142]. The few that decide to seek medical care are often seen by physicians that have received very little training on how to diagnose or report pesticide poisonings [69]. The result is a vast underestimate of the true scope of harm to this largely Latinx community. And because non-occupational injuries from pesticides are often compiled solely from reports to Poison Control Centers – utilization of which is known to be much lower for BIPOC and people of lower socioeconomic status [64–66] – a systemic issue exists with the underlying data that the program is built on.

An underfunded surveillance system that relies exclusively on a dataset that extensively underrepresents harm to BIPOC and lower-income communities is designed to fail. While the SENSOR-Pesticides program was built with the best of intentions, its failure to encompass all states and address the underlying deficiency of the data it uses has severely diminished its effectiveness.

In addition to reported incidents of pesticide harm, another line of evidence that can be used to assess the real-world consequences of a pesticide’s approval is epidemiology. One benefit of epidemiology over the typical *in vivo* toxicology studies done on animals is that epidemiological studies can give a regulatory body information about disparate impacts to specific populations of people that may be at higher risk. In fact many epidemiological studies, like the Center for the Health Assessment of Mothers and Children of Salinas (CHAMACOS) and Columbia Center of Children’s Environmental Health studies, were specifically designed for that purpose [80, 143].

Historically, epidemiological studies have not been accounted for or incorporated into EPA’s pesticide risk assessments and, therefore, had little impact on the agency’s overall decisions. With recommendations from the National Research Council of the National Academy of Science, EPA embarked on a process to incorporate epidemiology into its risk assessments that culminated in finalized guidance in 2016 [144]. In conjunction with a 2016 risk assessment of the pesticide chlorpyrifos that had partially incorporated epidemiological studies in a quantitative manner for the first time, this was seen as a major step forward for public health [145].

Yet despite these positive initial steps, intense lobbying and pressure from the pesticide industry has had a chilling effect on the agency’s use of epidemiology in its recent assessments [146]. The EPA’s pesticide office has continually failed to incorporate these studies in its quantitative risk assessments for pesticides – even those with robust epidemiological datasets, like paraquat, atrazine and 2,4-D [147–149]. And while chlorpyrifos was ultimately prohibited on food crops by the EPA in 2021 (following a court order), the agency reversed its initial 2016 decision to partially incorporate epidemiological studies into its quantitative risk assessment using dose reconstruction [150]. This was done in violation of a scientific advisory panel’s recommendations [151] and likely played a major role in allowing non-food uses of the pesticide to remain an ongoing threat to farmworkers and the general population.

By consistently analyzing incident numbers that are recognized to drastically underestimate the true scope of harm from pesticides – and continually failing to incorporate follow up epidemiological studies designed to uncover risks that were missed during the approval process – EPA is actively obstructing its own ability to respond to evidence of disparate impacts to BIPOC and communities of low-income and wealth.

#### Children lack necessary protections

The FQPA implemented an additional margin of safety meant to protect children, the most highly susceptible population to chemical poisons [89, 152]. This statutorily required safety margin came in the form of a default safety factor that would effectively reduce the amount of pesticide considered “safe” by 10-fold to account for the heightened susceptibility of young people who are still developing and growing (hereafter “FQPA children’s safety factor”). This was accompanied by a newly implemented aggregate assessment that directed EPA to assess non-occupational risk from multiple, combined exposure pathways, such as residential use and food exposures.

Widely lauded by the public health community, FQPA’s protections for children were strong, and EPA’s initial interpretation of the plain language of their statutory requirement was encouraging. In an early guidance document, EPA stipulated that it would err on the side of applying the FQPA children’s safety factor when there was scientific uncertainty about its necessity and even consider raising it in some cases [153].

Yet despite these positive initial steps, implementation of the FQPA children’s safety factor has been dismal from the outset. By 2001 EPA had only applied an extra margin of safety for children in 13 of 44 instances for organophosphate pesticides, and was chastised by its own watchdog agency in 2006 for primarily measuring its achievements under FQPA in terms of how often it met its registration deadlines rather than how it reduced risk to children [154, 155]. A review of 59 pesticides by the National Research Council found that EPA only implemented a FQPA children’s safety factor for 11 of them – with the full 10x margin of safety only being used for five [156]. A 2013 analysis by the United States Government Accountability Office (GAO) found that, out of 412 pesticide decisions, EPA retained the default 10x FQPA children’s safety factor only 22% of the time – it reduced the safety margin 75% of the time and increased it 3% [157]. A recent in-depth analysis of 47 non-organophosphate pesticides found that only 13% of acute food exposures and 12% of chronic food exposures incorporated any FQPA children’s safety factor whatsoever – and when it was included it was often in lieu of, not in combination with, a separate database uncertainty factor [158].

EPA’s justification for rarely incorporating the protective safety factor comes from the language of the law itself, which gives the EPA discretion to reduce the 10x FQPA children’s safety factor if such a determination can be made “…on the basis of reliable data…” [159]. Yet EPA’s current practice is such that the only time it retains the FQPA children’s safety factor is in the rare case where there is overtly severe developmental toxicity in rodent studies, on the level of serious structural malformations or death [156, 158]. When it decides to reduce or eliminate the FQPA children’s safety factor it is often based entirely on two or three rodent studies funded by the pesticide registrant, often conducted in the same laboratory [160].

Even in the few cases where EPA does incorporate a FQPA children’s safety factor, it is largely viewed as a moving target by the pesticide industry. Following a 2011 EPA decision to reduce the FQPA children’s safety factor for a class of pesticides called pyrethroids from 10x to 3x [161], a group of companies that sell pyrethroids developed a model that resulted in the complete elimination of the pyrethroid FQPA children’s safety factor in 2019 [162]. This happened even after multiple Scientific Advisory Panels found serious deficiencies with the model the registrants used [163, 164]. Ultimately, the consequences of such a move translated into the continued approval of uses of pyrethroids that would otherwise have been cancelled due to human health concerns – mainly those uses in people’s homes where exposures to children are often the highest.

Ultimately any disproportionate effects of pesticides on BIPOC or communities of low-income and wealth are going to be magnified even higher in their children because children will always be more susceptible to developmental toxins than adults [165, 166]. With 53% of migrant children having an unmet health need compared to 2.2% of all U.S. children, many BIPOC children may also have greater sensitivity to pesticides due to compounding stressors or other factors [165, 167, 168]. By using its discretion to overwhelmingly reduce protections for children instead of retaining them, EPA is perpetuating a system that propagates undue risk to lower-income children of color.

## How disproportionate pesticide impacts can be alleviated

The most consequential and important recommendation we have is for the USA to adopt the Precautionary Principle, which guides environmental policy in the European Union (EU) [169]. In fact, we believe it is impossible to truly “solve” this environmental injustice in the context of our current system, which masquerades as scientific norm in a country that has consistently normalized oppression to people of color. It is this system that attempts to monetize people’s lives and well-being, attempting to determine whether any resulting harm from the action is “worth it” (with the implicit message that some people’s lives are not worth as much as others). It is a system that unduly benefits the entrenched, capitalist agrochemical regime by consistently prioritizing powerful economic markets at the expense of people’s lives and well-being.

However, under the Precautionary Principle we ask “How little harm is possible” rather than “How much harm is allowable” [170]. Being proactive instead of reactive, it’s a simple change of perspective that can mean the difference of life or death to countless BIPOC and people living in poverty. Use of the Precautionary Principle can be compatible with a thriving agricultural sector, as evidenced by the EU’s incredibly high export value of agricultural commodities [171]. The Precautionary Principle is often derided as “extreme” and “radical” by those in the USA profiting from the current, broken system. However, the very fact that it is considered “extreme” or “radical” to ensure that everyone has the right to a healthy environment and life further proves to us just how unjust our current system is.

Given the realities of today, we fully acknowledge that this paradigm change within FIFRA itself is likely unattainable in the near term. While the Precautionary Principle should be the ultimate goal, advocating for a more just system also includes making an unjust system better. Below we lay out seven Actions that can, and should, be implemented immediately to reduce pesticide harm to BIPOC and low-income communities in the USA and beyond.

### Action #1 – eliminate or reduce the pesticide safety double standard

Any double standard for different groups of people is unacceptable when it comes to protections from harmful pollutants. The confluence of three different pesticide laws (FIFRA, FFDCA and FQPA) to exclude a largely Latinx farmworker population from protections that everybody else is afforded stands today as one of the most overtly racist aspects of current pesticide law. The clear response to this should be to amend FQPA or FIFRA to ensure that the “reasonable certainty that no harm will result” safety threshold be extended to include those exposed to pesticides through their work. This is the current safety threshold that must already be met for those exposed to pesticides through multiple pathways, including their diet and other non-occupational exposures.

Absent a legislative fix, there are things EPA can do right now within its current authority to reduce the protection gap between farmworkers and the general public. The first is to immediately implement the entirety of EPA’s 2009 guidance document “Revised Risk Assessment Methods for Workers, Children of Workers in Agricultural Fields, and Pesticides with No Food Uses” [92].

However, this alone is not enough.

The second thing EPA should do under its current authority is to finally, and formally, define “no unreasonable adverse effects” in a way that appropriately recognizes and reduces harm to agricultural workers. Since 1972, the core statutory requirement of FIFRA has been for EPA to balance the costs and benefits when deciding whether to approve a pesticide. However, this has never been done transparently and amounts to more of a subjective exercise subject to the whims of political pressure, undue influence, and a culture that makes it difficult to say “no.” Defining what types of harms are not acceptable to workers by setting forth clear standards would help ensure that that the EPA cannot generically allow the harms to workers be outweighed by the purported benefits of a pesticide in the agency’s registration decisions.

### Action #2 – implement a system to adequately monitor and account for harms to environmental justice communities

While the SENSOR-Pesticides program is better than nothing, it is wholly inadequate to monitor and surveil harm from pesticides to environmental justice communities in the USA. We must develop a well-funded, nationwide monitoring system to incorporate data from *all* states and standardize reporting and collection to the federal government. This national monitoring and surveillance system must incorporate occupational *and* non-occupational harm.

However, without addressing the inherent issues that lead to underreporting, any national system is destined to fail in its purpose. The federal government must also implement measures to reduce incident underreporting, particularly in BIPOC and low-income communities. This could include things like requiring employers to report incidents or face steep fines (similar to what is proposed in the “Protect America’s Children from Toxic Pesticides Act” (PACTPA) [172]), educating clinicians on how to diagnose and report pesticide poisoning, explicitly requiring public schools and other federally-funded facilities that use pesticides to report incidents, and allowing for anonymous reporting from those who might fear retaliation.

Just as important, EPA must implement a regulatory framework that is inclusive and not dismissive of epidemiological data. Current guidance and practice are simply unacceptable. Particularly as the agency moves away from reliance on *in vivo* animal experiments [173], human epidemiology – done by independent researchers with a lens towards marginalized communities – must play a larger role in EPA’s registration decisions. Above all, this will require that the agency stand up to the pesticide industry instead of cowering to it.

### Action #3 – strengthen worker protections

EPA must require medical monitoring for those who work occupationally with pesticides, as is common for most other occupations that work closely with dangerous chemicals. This can be done immediately for organophosphates and carbamates following the framework implemented in Washington and California [101, 102]. However, only monitoring these two classes is not sufficient. EPA can and should require pesticide registrants to supply a clinical test capable of confirming a pesticide overexposure from their products via its authority under FIFRA section 6(a)(2) for any pesticide or pesticide class implicated in worker harm. This would significantly improve access to health care for farmworkers, aid in Workers’ Compensation claims and reduce harmful exposures. This would also aid in achieving Action #2.

The importance of the pesticide label to the safe use of a pesticide cannot be understated. Given the widespread use of pesticides by non-English speakers in the USA, the fact that pesticide labels are only required to be provided in English is entirely unacceptable. The EPA has the clear authority to mandate labels be provided in languages other than English in order to protect the public [174]. The agency should mandate, at a minimum, that all pesticide labels immediately be provided in the Spanish language. Ultimately, along the lines of what is proposed in PACTPA [172], EPA should strive to require pesticide labels be provided in any language where information exists that at least 500 people who speak that same language use a particular pesticide product.

### Action #4 – reduce unintended pesticide harms

The more complex the pesticide label and the more restrictions put in place to protect people or the environment, the higher likelihood that there will be unintended pesticide uses that can result in serious harms. The *practicality* of label restrictions for both agriculture and residential use must become an integral part of the registration decision. This is completely unaccounted for in current pesticide approval decisions. Such an approach will require data on label compliance and noncompliance to give the agency information about what restrictions/mitigations are commonly followed and which are not. This approach would be guided by data and science instead of the current approach, which is based solely on the incorrect assumption that all labels can and will be followed 100% by everyone.

By engaging with the farmworker community, EPA can also identify ways to strengthen training requirements for workers in ways that are engaging and the information more likely to be retained. EPA must publicly commit to implementing reasonable requirements in a timely fashion based on input and meetings with farmworkers and their representatives.

Perhaps most important is for the EPA to strictly enforce all existing requirements in the Worker Protection Standard. This would require appropriating resources for inspection and enforcement activities and holding unscrupulous employers accountable to the full extent possible under the law.

### Action #5 – adequately protect those most vulnerable to pesticide harm – children

EPA should fully incorporate the 10x FQPA children’s safety factor across the board for all pesticides when analyzing harm to children, and increase it when data indicate that greater safety buffer is needed. We recognize that EPA has the discretion to reduce or eliminate the FQPA children’s safety factor if it so chooses, but this exception has swallowed the rule. Sometimes the agency does have studies in its possession that can be interpreted to imply that a safety buffer is not necessary; however, in practice, every decision to reduce the FQPA children’s safety factor is made under an enormous amount of scientific uncertainty. Often only a few studies done in the same laboratory and funded by the pesticide companies are available for review, or certain peer reviewed studies or epidemiological studies are ignored or discounted in some manner. This would meet very few scientists’ definition of “reliable data,” yet that is the statutory definition of the data EPA uses when opting to eliminate the FQPA children’s safety factor. Pesticide companies are even combining their resources to form separate corporate entities with the sole intent to “address” the FQPA children’s safety factor for their products – and have been successful in eliminating these protections [162, 175]. Rarely, if ever, is there any instance when an abundance of research from multiple different labs without a financial conflict of interest all find that young children or the developing fetus are not more susceptible to pesticide poisoning than an adult. Yet eliminating the FQPA children’s safety factor is the norm, not the exception.

We propose a regulatory rethinking of what the FQPA children’s safety factor represents and an acknowledgement that its intended purpose when Congress proposed it was not for EPA to regularly cast it aside. While all children are more susceptible to pesticide harm than adults, some children – particularly BIPOC and those in low-income or low-wealth families – will often carry a higher burden of exposure [165, 166]. Widespread utilization of the FQPA children’s safety factor is one way to protect this subpopulation of the most vulnerable of the vulnerable. EPA has an enormous opportunity with its discretion under current law to immediately put in place greater protections aimed at preventing harm to the next generation – implementing the FQPA children’s safety factor across the board is one easy way to accomplish this.

### Action #6 – prohibit export of unregistered pesticides to other countries

Current law allows for the export of pesticides that are not registered in the USA – even those that have been banned here due to human health or environmental harms. This practice must end. The most harmful of these prohibited pesticides are largely going to lower income countries that have higher rates of pesticide poisonings (Fig. 4 and Additional file 1). If a pesticide has not met our standards for safety, we should not actively provide it to other countries that have even fewer protections and safeguards than we do. To do so makes us complicit in any harm that it causes. The European Commission has already begun implementing this moral imperative in the EU [37].

The USA must also ratify the Rotterdam and Stockholm Conventions. The USA is a signatory on both treaties, however we remain one of the few countries left in the world that has not ratified either [176, 177]. That puts us in a gray area for compliance purposes. Some of the extremely hazardous pesticides we’ve exported in recent years – like alachlor, carbofuran and phorate – are listed in Annex III of the Rotterdam convention and subject to Prior Informed Consent (PIC), which is a mechanism by which countries can opt out of receiving harmful chemicals through trade [178]. The USA has even violated this international treaty as recently as two years ago by exporting carbofuran to the African country of Mauritius in 2019 after the country specifically informed the Rotterdam Committee in 2018 that it does not consent to carbofuran imports [179, 180].

### Action #7 – assess and rectify regulatory capture within the EPA pesticide office

The pesticide office at EPA is plagued by an enormous amount of chemical industry influence [181, 182]. There are many reasons for this, but the end result is the same – industry interests are often put above public health interests and harmful products stay on the market. A culture such as this is incompatible with environmental justice and scientific integrity. This makes it difficult for EPA to implement changes that positively affect disenfranchised and marginalized communities and will always be an impediment to true change within the agency.

We believe a third-party audit of how EPA’s operating procedures and management practices allow for undue industry influence and what effects it has on environmental justice communities is long overdue. The National Research Council is one example of an independent party that could study this matter and report back to EPA on recommended strategies to further separate the regulators from the regulated in a manner that would benefit BIPOC and low-income communities and, by extension, the broader public.

Shifting the culture in the EPA’s pesticide office is critical to ensuring that any measures taken to reduce the disproportionate impacts on environmental justice communities are realized.

## Conclusions

Exposure to many, if not most, pollutants fall along racial, ethnic, or sociodemographic lines in the USA – and pesticides are no exception. Disparities in exposure and harm from pesticides are widespread, impacting BIPOC and low-income communities in both the rural and urban settings and occurring throughout the entire lifecycle of the pesticide from production to end-use. The root causes of these disparities involve hundreds of years of systematic oppression kept in place through structural racism and classism in the USA. Despite many of the atrocities that gave rise to these disparities being seemingly in the past, there are ways in which the federal government perpetuates these disparities and hinders progress even today. Here we’ve identified laws and regulatory practices and policies that allow for such disparities to remain entrenched in everyday life for environmental justice communities. While the true fix is to shift the USA to a more just system of preventing pollution exposure to everyone regardless of skin tone or income, there are actions that can be taken right now to make our unjust regulatory system work better for everyone and begin to rectify the grave injustices it has perpetuated.

## Supplementary Information

**Additional file 1: Supplemental Methods.** This file contains the methodology used for the literature review and the data collection for the Figures and Table in the manuscript.

## Data Availability

All data generated or analyzed during this study are publicly available at the cited links.

## Abbreviations

BIPOC: Black, Indigenous and People of Color
CAA: Clean Air Act
CDC: U.S. Centers for Disease Control and Prevention
CHAMACOS: Center for the Health Assessment of Mothers and Children of Salinas
CWA: Clean Water Act
ECHO: Enforcement and Compliance History Online Database
EPA: United States Environmental Protection Agency
EPA-OIG: EPA Office of the Inspector General
EU: European Union
FFDCA: Federal Food, Drug and Cosmetic Act
FIFRA: Federal Insecticide, Fungicide, and Rodenticide Act
FQPA: Food Quality Protection Act of 1996
GAO: United States Government Accountability Office
GNI: Gross National Income
LMIC: Low-to-middle income country
NAICS: North American Industry Classification System
OC: Organochlorine
OECD: Organisation for Economic Co-operation and Development
OP: Organophosphate
OSHA: U.S. Occupational Safety and Health Administration
PACTPA: Protect America’s Children from Toxic Pesticides Act
PIC: Prior Informed Consent
PPE: Personal Protective Equipment
RCRA: Resource Conservation and Recovery Act
SENSOR: Sentinel Event Notification System for Occupational Risk
TEK: Traditional Ecological Knowledge
UAPP: Unintentional, acute pesticide poisoning
WPS: Worker Protection Standard

## Footnotes

1 While no other analyses are currently undertaken, EPA does state that: “Further considerations are also currently in development as [EPA’s Office of Pesticide Programs] has committed resources and expertise to the development of specialized software and models that consider exposure to other types of possible bystander exposures and farm workers as well as lifestyle and traditional dietary patterns among specific subgroups.” See references [119–121].
