# Supplementary material for: Pesticides and environmental injustice in the USA: root causes, current regulatory reinforcement and a path forward
Source: BMC Public Health. 2022 Apr 19;22:708. doi: 10.1186/s12889-022-13057-4 (PMC9017009; doi:10.1186/s12889-022-13057-4)
Supplement: Supplementary file 1 — Additional file 1: Supplemental Methods. This file contains the methodology used for the literature review and the data collection for the Figures and Table in the manuscript. [file 12889_2022_13057_MOESM1_ESM.docx]

**Supplemental Methods**

Literature Review

Studies on pesticide disparities to low-income communities and people of color were compiled using a purposive sampling technique, whereby studies were quickly assessed for relevance using the authors’ professional judgement and knowledge of the subject matter to manageably compile relevant literature on a wide-ranging subject matter.

Briefly, Google Scholar was searched using the keyword “pesticide” in conjunction with various other keywords, such as “race, “racial,” “ethnic,” “ethnicity,” “poverty,” “income,” and “demographics.” Given the genericity of these keywords, most identified studies were irrelevant to our review. Our inclusion criteria were: 1) The study was conducted on a population in the USA, and 2) The study analyze exposure to, or harm from, a pesticide ingredient to a population that does not identify as non-Hispanic, white and compared it to a population that identified as non-Hispanic, white. Further sources were identified via the authors’ involvement or prior knowledge of the study. All studies that met our search criteria were further mined for relevant references in their respective bibliographies. The latter approach was crucial because information about race, ethnicity, sociodemographics and type of chemical was often implicit in the study or buried in the main text and difficult to identify in a database search.

Our purposive sampling technique was designed to quickly, qualitatively and manageably identify important studies that represented all the different racial, ethnic or socioeconomic subgroups and their disparate exposure to, or harm from, pesticides. Consistent with the objective of our study, we were interested in identifying the broadest range of exposure scenarios and demographic subgroups. For instance, most of the relevant literature we found in our review concerned farmworkers exposed to pesticides occupationally, therefore special consideration was given to ensure that studies on non-occupational exposures, manufacturing exposures and other demographic groups were not missed during our literature review.

Therefore, our search strategy was not intended to be inclusive of every single study of relevance, but geared more towards maximum variation sampling (i.e. identifying whether disparate exposures and harms from pesticides was a consistent finding across different exposure scenarios and demographic subgroups).

Figure 1

On November 4, 2021, the Facility Search tool in EPA’s Enforcement and Compliance History Online (ECHO) database was utilized to identify all “active/operating” facilities in the USA with a North American Industry Classification System (NAICS) code of “32532” [1]. This code designates a facility as “Pesticide and Other Agricultural Chemical Manufacturing” and should be inclusive of all facilities that manufacture pesticides in the USA. Of the 581 facilities identified in the USA, there were 31 facilities with "Significant Violations" – defined as one or more of the following: High Priority Violation (Clean Air Act), Significant/Category I Noncompliance (Clean Water Act), Significant Noncomplier (Resource Conservation and Recovery Act), or Serious Violator (Safe Drinking Water Act). The “Detailed Facility Report” was accessed on ECHO for each of the 31 facilities with Significant Violations, and information on the surrounding 1-mile demographic profile was extracted and included in Figure 1.

“% BIPOC” 1 mile from the facility is the same value as the “Percent People of Color” statistic from the demographic output in each facility’s Detailed Facility Report from the ECHO database. ECHO defines the “Percent People of Color” as “the percentage of the population of the given area that consists of people of color, based on the 2010 U.S. Census. The field is calculated by subtracting the number of persons who are white (and not of Hispanic origin) from the total persons. This number is then divided by the total persons and multiplied by 100 to determine the percentage” [2].

National “% BIPOC” was calculated by subtracting the number of people who identify as “White alone, not Hispanic or Latino” from the total population based on US census estimates for 2019 [3]. This number was divided by the total population, multiplied by 100 and rounded to the nearest whole number. State “% BIPOC” was calculated by subtracting the number of people who identify as “Not Hispanic” and “One Race: White” in each state from the total state population based on US census estimates for 2019 [4]. This number was divided by the total population, multiplied by 100 and rounded to the nearest whole number.

“% low income” 1 mile from the facility is the same value as the “Percent with Low Income” statistic from the demographic output in each facility’s Detailed Facility Report from the ECHO database. ECHO defines the “Percent with Low Income” as “The percent of people in the selected area that have an income less than two times the poverty level, based on the 2014-2018 ACS 5-Year Summary” [2].

State and National “% low income” were calculated by dividing the number of people living below 200% of the federal poverty level in the USA or in each state by the total number of people for whom poverty status was determined based on US census data for 2020 [5]. This number was then multiplied by 100 and rounded to the nearest whole number.

For calculating averages 1 mi. from the facility, percentages were averaged within each state before averaging between states. This was done so that each state would only be counted once in calculating relevant state averages.

Figures 2 and 3

Data on urinary levels of all pesticides except organochlorine (OC)/legacy pesticides were compiled from the U.S. Centers for Disease Control and Prevention’s (CDC’s) Fourth National Report on Human Exposure to Environmental Chemicals Updated Tables, January 2019, Volume One [6]. Data on serum levels of OC/legacy pesticides were compiled from CDC’s Fourth National Report on Human Exposure to Environmental Chemicals Updated Tables, January 2019, Volume Two [7]. Appendix C in each of these reports was used to identify which pesticides and metabolites were analyzed by CDC in urinary and serum samples. Many pesticides/metabolites were not detected at levels above the limit of detection and could not be included in this paper. If a geometric mean or 95^th^ percentile value was identified for the total population and the three surveyed demographic groups (non-Hispanic whites, non-Hispanic Blacks, and Mexican Americans) in the same survey year, it was included in Figures 2 or 3. If a mean or 95^th^ percentile value for a pesticide/metabolite was identified for all groups in multiple survey years between 1999-2016, the values from different years were averaged for each group.

For urinary pesticide levels, CDC reports pesticide concentrations per volume of urine and a separate creatinine-adjusted concentration to account for variations in urine dilution. Because our primary objective was to compare pesticide concentrations across different racial/ethnic subgroups, we chose to use non-creatinine-adjusted urinary concentrations for our analysis. The reason being that race/ethnicity is a strong predictor for urinary creatinine concentrations, which will ultimately bias creatinine-adjusted results when comparing across these demographic groups [8, 9]. Therefore, one limitation of our analysis is that it did not account for potential differences in urinary dilution for some of the measured pesticides/metabolites.

Figure 4

A list of all pesticide exports reported to the EPA under FIFRA 17(a)(2) for the years 2015-2019 was requested under the Freedom of Information Act and produced to us by the agency on January 11, 2021 [10]. This information provided 1) the name of the exporting company, 2) product name, 3) active ingredient(s) name and CAS #, and 4) the destination country of every pesticide exported from the USA between 2015-2019 that was not registered for use in the USA. EPA only provided information on export to the “European Union” and not which individual countries within the European Union received the import. The agency did not provide information on exported volumes because these data are considered confidential under section 7 of FIFRA.

From this list, we focused on the products containing any organophosphate (OP) or carbamate active ingredient. A list of carbamate and organophosphate active ingredients from Alan Wood’s “Compendium of Pesticide Common Names” [11] was cross-referenced with the active ingredients reported to EPA under FIFRA 17(a)(2) to identify a total of 734 unique listings of OP/carbamate product exports that were reported to the EPA between 2015-2019. These products contained a total of 26 OP or carbamate active ingredients.^[[1]](#footnote-1)^ During 2015-2019, 53 nations received imports of products containing these 26 organophosphate or carbamate pesticides from the USA.^[[2]](#footnote-2)^

Of these 26 organophosphate or carbamate active ingredients, eight have no active registration for use in the USA according to EPA’s Pesticide Product and Label System [12].^[[3]](#footnote-3)^ During 2015-2019, 42 nations received imports of products containing these eight OP or carbamate pesticides from the USA.^[[4]](#footnote-4)^

Low and Middle-Income countries (LMICs) were identified based on The Organisation for Economic Co-operation and Development’s (OECDs) determination that the country was determined to be a LMIC by the World Bank and eligible to receive official development assistance [13]. Any country or territory not considered “Least Developed,” “Other Low Income,” “Lower Middle Income,” or “Upper Middle Income” was assumed to be “High Income.” Since info on export to the European Union was not divided up by country, the European Union was considered “High Income” for this analysis.

Information on percent Unintentional, Acute Pesticide Poisoning (% UAPP) was extracted from Boedeker et al, 2020 [14]. Each country’s % UAPP – which is the percent of agricultural workers in each country estimated to experience an unintentional poisoning each year – was identified from Table 10 in Boedeker et al. If available, each country in our analysis was assigned a % UAPP based on data compiled from that specific country. If the specific country was not included in Table 10, then the mean % UAPP or % UAPP for a representative country in the available subregion was used instead. Tajikistan, Uzbekistan, and the European Union were not given % UAPP values and omitted from our analysis because no UAPP estimates were available for the “Central Asia” subregion or the European Union as a whole.

Table 1

Data on the Worker Protection Standard (WPS) compliance and enforcement was extracted from EPA’s ECHO database. On December 3, 2021, ECHO’s Pesticide Dashboard (also called the Worker Protection Standard (WPS) Dashboard) was accessed, which compiles data on facilities subject to the WPS requirements, and EPA, State and Tribal actions with regards to those facilities [15]. EPA estimates that 304,106 facilities in the USA are pesticide-using agricultural establishments that hire workers and are subject to WPS requirements; and that this number is representative of most years [16]. We therefore assumed that there were 304,106 agricultural operations subject to the WPS in each of the years we analyzed. Data from the most recent five years available (2015-2019) were compiled for the Table and included the number of facility inspections, the number of violations identified, and the number of enforcement actions taken by State, Federal and Tribal governments. Enforcement actions that were categorized as “Warnings Issued” in the ECHO database were combined with the number of violations where no action was taken to populate the “No Action/Only Warning” column in the Table. All other enforcement actions in the ECHO database categorized as “Admin. Hearings/Civil,” “Sent to EPA,” “Additional Actions,” “Stop Sale, Seizure, Quarant,” and “Cases with Fines” were combined and included in the “Enforcement Action Taken” column in the Table. The “Inspection Rate” was calculated by dividing the “Facilities Inspected” by the “Total WPS Facilities.” The “Violation Rate” was calculated by dividing the “Violations Found” by the “Facilities Inspected.” The “Enforcement Rate” was calculated by dividing the “Enforcement Action Taken” by the “Violations Found.”

**References**

1. U.S. EPA. Enforcement and Compliance History Online (ECHO) Database. Facility Search – Enforcement and Compliance Data. https://echo.epa.gov/facilities/facility-search. Accessed 4 Nov 2021.

2. U.S. EPA. Enforcement and Compliance History Online (ECHO) Database. Detailed Facility Report Data Dictionary. https://echo.epa.gov/help/reports/dfr-data-dictionary. Accessed 4 Nov 2021.

3. U.S. Census Bureau. American Community Survey 1-Year Estimates Detailed Tables - Total Population. Hispanic Or Latino Origin By Race. Table ID: B03002. 2019. https://data.census.gov/cedsci/table?q=HISPANIC%20OR%20LATINO%20ORIGIN%20BY%20RACE&tid=ACSDT1Y2019.B03002. Accessed 4 Nov 2021.

4. U.S. Census Bureau. State Population by Characteristics: 2010-2019. Annual Estimates of the Resident Population by Sex, Race, and Hispanic Origin: April 1, 2010 to July 1, 2019 by State. https://www.census.gov/data/tables/time-series/demo/popest/2010s-state-detail.html. Accessed 4 Nov 2021.

5. U.S. Census Bureau. Current Population Survey (CPS) Annual Social and Economic (ASEC) Supplement. POV-46. Poverty Status by State. Poverty status in 2020. https://www.census.gov/data/tables/time-series/demo/income-poverty/cps-pov/pov-46.html. Accessed 4 Nov 2021.

6. U.S. Centers for Disease Control and Prevention. Fourth National Report on Human Exposure to Environmental Chemicals, Updated Tables, January 2019, Volume One. 2019.

7. U.S. Centers for Disease Control and Prevention. Fourth National Report on Human Exposure to Environmental Chemicals, Updated Tables, January 2019, Volume Two. 2019.

8. Barr DB, Wilder LC, Caudill SP, Gonzalez AJ, Needham LL, Pirkle JL. Urinary Creatinine Concentrations in the U.S. Population: Implications for Urinary Biologic Monitoring Measurements. Environmental Health Perspectives. 2005;113:192–200.

9. Kuiper JR, O’Brien KM, Ferguson KK, Buckley JP. Urinary specific gravity measures in the U.S. population: Implications for the adjustment of non-persistent chemical urinary biomarker data. Environment International. 2021;156:106656.

10. U.S. EPA. FOIA response to the Center for Biological Diversity regarding FIFRA 17(a)(2) Exports. January 11, 2021. Tracking number: EPA-2021-000860.

11. Wood A. Compendium of Pesticide Common Names. https://pesticidecompendium.bcpc.org/. Accessed 4 Nov 2021.

12. U.S. EPA. Pesticide Product and Label System database. Version: 2.4.1.1. https://ordspub.epa.gov/ords/pesticides/f?p=PPLS:1. Accessed 21 Jun 2021.

13. Organisation for Economic Co-operation and Development. DAC List of ODA Recipients. Effective for reporting on 2020 flows. https://www.oecd.org/dac/financing-sustainable-development/development-finance-standards/DAC-List-of-ODA-Recipients-for-reporting-2020-flows.pdf. Accessed 1 Jun 2021.

14. Boedeker W, Watts M, Clausing P, Marquez E. The global distribution of acute unintentional pesticide poisoning: estimations based on a systematic review. BMC Public Health. 2020;20:1875.

15. U.S. EPA. Enforcement and Compliance History Online (ECHO) Database. Analyze Trends: Pesticide Dashboard. https://echo.epa.gov/trends/comparative-maps-dashboards/state-pest-dashboard?state=National. Accessed 3 Dec 2021.

16. U.S. EPA. Enforcement and Compliance History Online (ECHO) Database. Pesticide Dashboard Help. Worker Protection Standard (WPS) Dashboard. https://echo.epa.gov/help/pesticide-dashboard-help. Accessed 3 Dec 2021.

1. Acephate, cadusafos, carbaryl, carbofuran, carbosulfan, chlorpyrifos, chlorpyrifos-methyl, diazinon, dichlorvos, dicrotophos, ethion, ethoprop, malathion, methomyl, mevinphos, naled, oxamyl, phorate, phosmet, profenofos, propoxur, prothiofos, tebupirimfos, terbufos, temephos, thiodicarb. [↑](#footnote-ref-1)
2. Algeria, Argentina, Australia, Bangladesh, Belize, Brazil, Canada, Chile, China, Colombia, Costa Rica, Cote d´Ivoire, Dominican Republic, Ecuador, Egypt, Ethiopia, European Union, Ghana, Guatemala, Haiti, Honduras, India, Indonesia, Iraq, Jamaica, Japan, Jordan, Kenya, Lebanon, Libya, Mauritius, Mexico, Morocco, New Zealand, Nicaragua, Nigeria, Pakistan, Panama, Peru, Philippines, Saudi Arabia, Senegal, South Africa, South Korea, Taiwan, Tajikistan, Thailand, Turkey, United Arab Emirates, Uruguay, Uzbekistan, Venezuela, Vietnam. [↑](#footnote-ref-2)
3. Cadusafos, carbofuran, carbosulfan, ethion, mevinphos, profenofos, prothiofos, temephos. [↑](#footnote-ref-3)
4. Algeria, Argentina, Australia, Bangladesh, Brazil, Chile, Colombia, Costa Rica, Cote d´Ivoire, Dominican Republic, Ecuador, Egypt, Ethiopia, European Union, Ghana, Guatemala, Haiti, Honduras, India, Indonesia, Iraq, Jordan, Kenya, Lebanon, Libya, Mauritius, Mexico, Morocco, Nicaragua, Pakistan, Panama, Peru, Philippines, Saudi Arabia, Senegal, South Africa, South Korea, Taiwan, United Arab Emirates, Uruguay, Uzbekistan, Venezuela. [↑](#footnote-ref-4)
